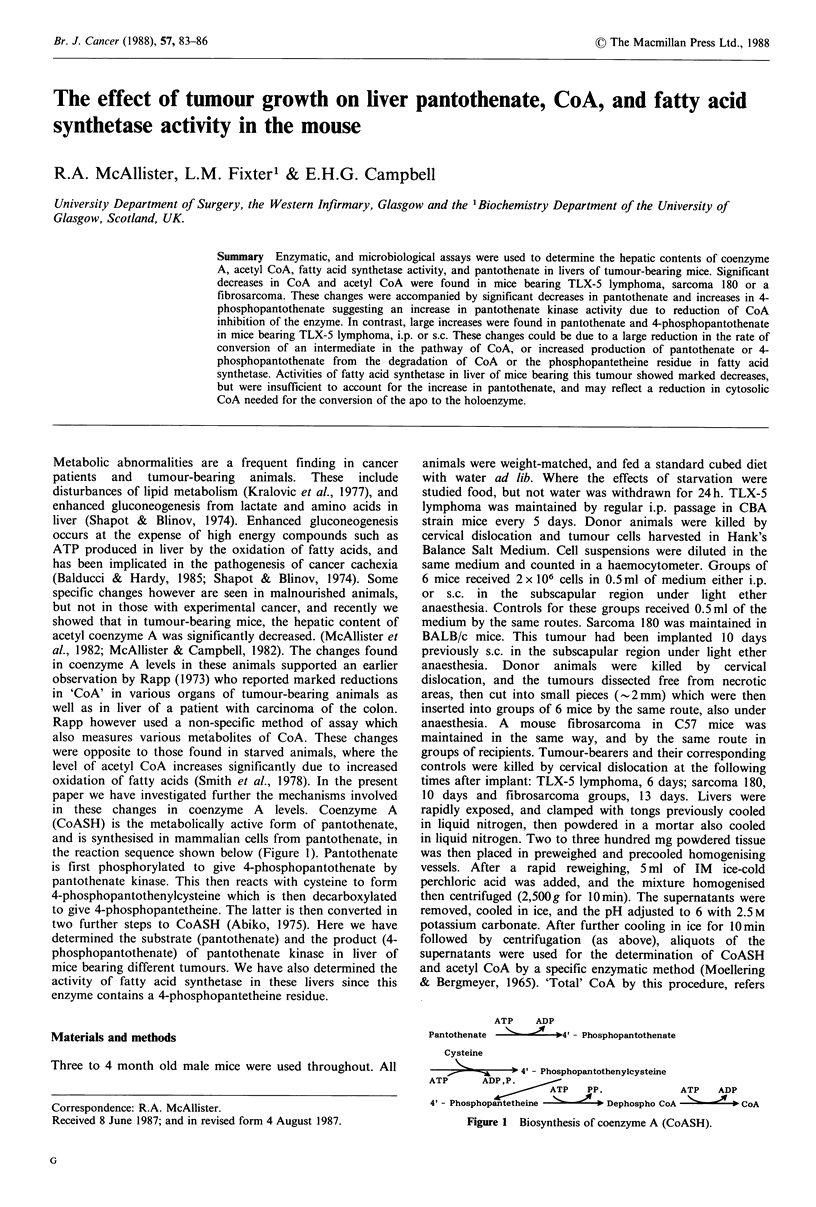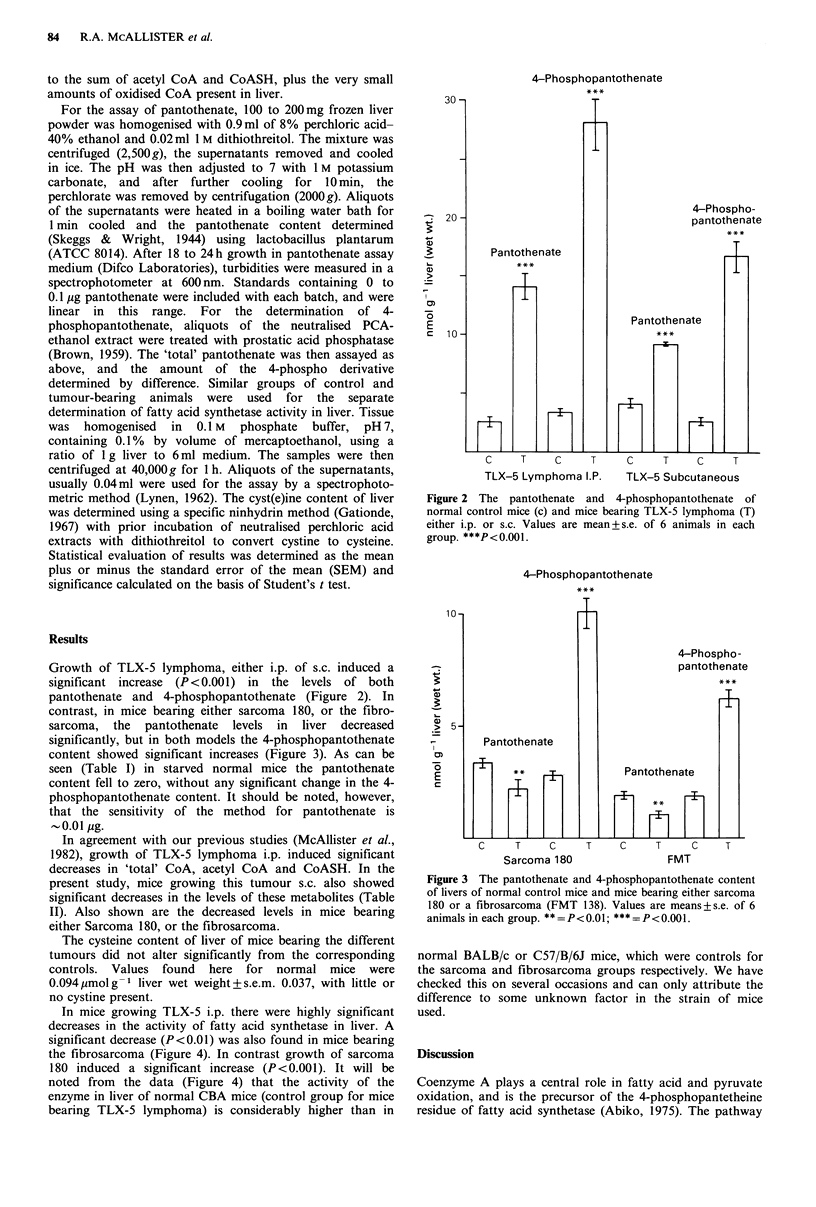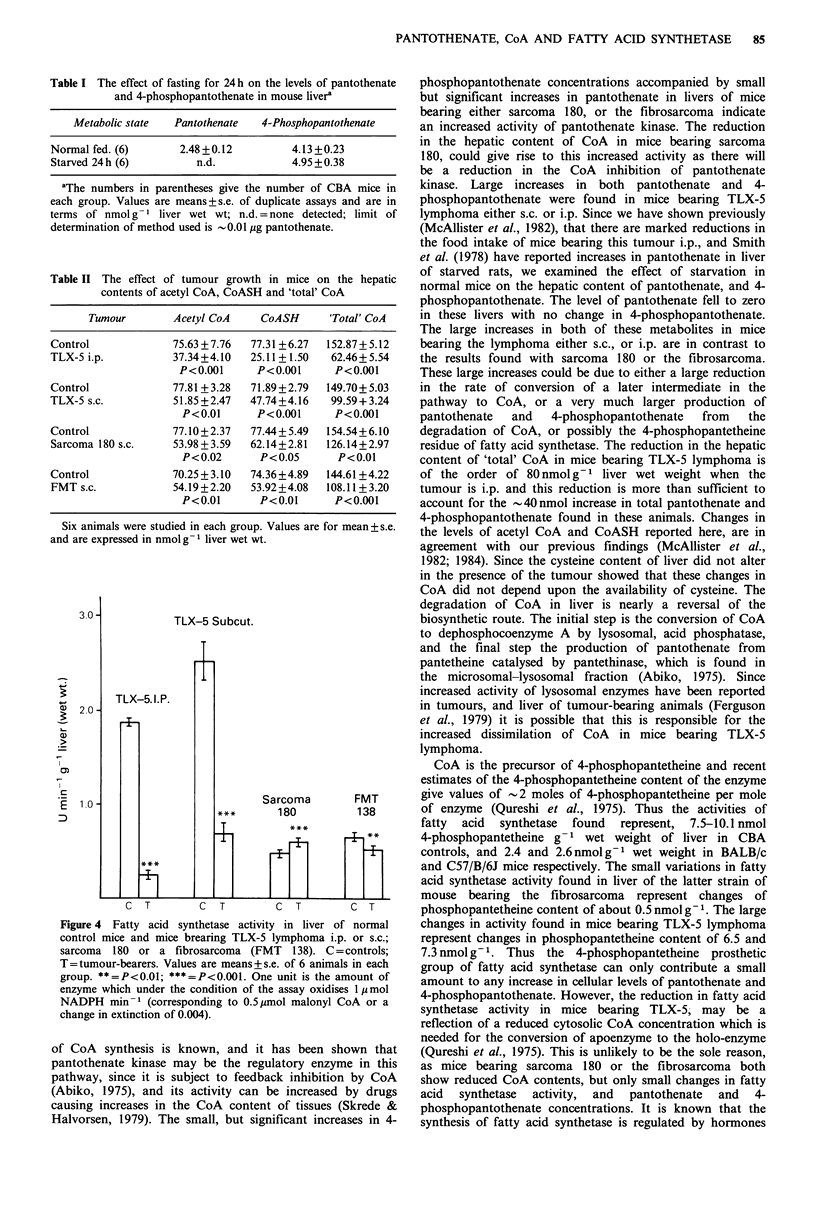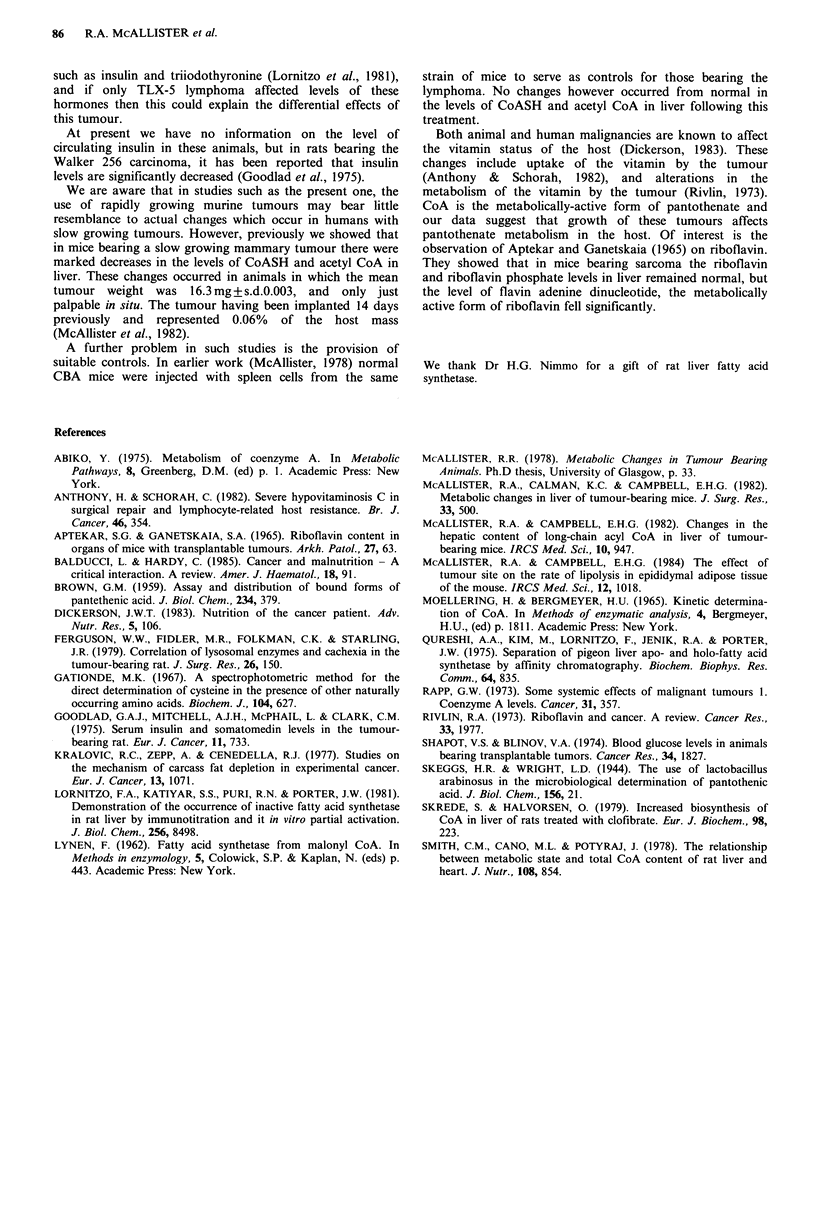# The effect of tumour growth on liver pantothenate, CoA, and fatty acid synthetase activity in the mouse.

**DOI:** 10.1038/bjc.1988.14

**Published:** 1988-01

**Authors:** R. A. McAllister, L. M. Fixter, E. H. Campbell

**Affiliations:** University Department of Surgery, Western Infirmary, Glasgow, Scotland, UK.

## Abstract

Enzymatic, and microbiological assays were used to determine the hepatic contents of coenzyme A, acetyl CoA, fatty acid synthetase activity, and pantothenate in livers of tumour-bearing mice. Significant decreases in CoA and acetyl CoA were found in mice bearing TLX-5 lymphoma, sarcoma 180 or a fibrosarcoma. These changes were accompanied by significant decreases in pantothenate and increases in 4-phosphopantothenate suggesting an increase in pantothenate kinase activity due to reduction of CoA inhibition of the enzyme. In contrast, large increases were found in pantothenate and 4-phosphopantothenate in mice bearing TLX-5 lymphoma, i.p. or s.c. These changes could be due to a large reduction in the rate of conversion of an intermediate in the pathway of CoA, or increased production of pantothenate or 4-phosphopantothenate from the degradation of CoA or the phosphopantetheine residue in fatty acid synthetase. Activities of fatty acid synthetase in liver of mice bearing this tumour showed marked decreases, but were insufficient to account for the increase in pantothenate, and may reflect a reduction in cytosolic CoA needed for the conversion of the apo to the holoenzyme.


Br. J. Cancer (1988), 57, 83–86 © The Macmillan Press Ltd., 1988

### The effect of tumour growth on liver pantothenate, CoA, and fatty acid synthetase activity in the mouse

R.A. McAllister, L.M. Fixter[1] & E.H.G. Campbell

*University Department of Surgery, the Western Infirmary, Glasgow and the [1]Biochemistry Department of the University of Glasgow, Scotland, UK.*

**Summary** Enzymatic, and microbiological assays were used to determine the hepatic contents of coenzyme A, acetyl CoA, fatty acid synthetase activity, and pantothenate in livers of tumour-bearing mice. Significant decreases in CoA and acetyl CoA were found in mice bearing TLX-5 lymphoma, sarcoma 180 or a fibrosarcoma. These changes were accompanied by significant decreases in pantothenate and increases in 4-phosphopantothenate suggesting an increase in pantothenate kinase activity due to reduction of CoA inhibition of the enzyme. In contrast, large increases were found in pantothenate and 4-phosphopantothenate in mice bearing TLX-5 lymphoma, i.p. or s.c. These changes could be due to a large reduction in the rate of conversion of an intermediate in the pathway of CoA, or increased production of pantothenate or 4-phosphopantothenate from the degradation of CoA or the phosphopantetheine residue in fatty acid synthetase. Activities of fatty acid synthetase in liver of mice bearing this tumour showed marked decreases, but were insufficient to account for the increase in pantothenate, and may reflect a reduction in cytosolic CoA needed for the conversion of the apo to the holoenzyme.

Metabolic abnormalities are a frequent finding in cancer patients and tumour-bearing animals. These include disturbances of lipid metabolism (Kralovic *et al.*, 1977), and enhanced gluconeogenesis from lactate and amino acids in liver (Shapot & Blinov, 1974). Enhanced gluconeogenesis occurs at the expense of high energy compounds such as ATP produced in liver by the oxidation of fatty acids, and has been implicated in the pathogenesis of cancer cachexia (Balducci & Hardy, 1985; Shapot & Blinov, 1974). Some specific changes however are seen in malnourished animals, but not in those with experimental cancer, and recently we showed that in tumour-bearing mice, the hepatic content of acetyl coenzyme A was significantly decreased. (McAllister *et al.*, 1982; McAllister & Campbell, 1982). The changes found in coenzyme A levels in these animals supported an earlier observation by Rapp (1973) who reported marked reductions in 'CoA' in various organs of tumour-bearing animals as well as in liver of a patient with carcinoma of the colon. Rapp however used a non-specific method of assay which also measures various metabolites of CoA. These changes were opposite to those found in starved animals, where the level of acetyl CoA increases significantly due to increased oxidation of fatty acids (Smith *et al.*, 1978). In the present paper we have investigated further the mechanisms involved in these changes in coenzyme A levels. Coenzyme A (CoASH) is the metabolically active form of pantothenate, and is synthesised in mammalian cells from pantothenate, in the reaction sequence shown below (Figure 1). Pantothenate is first phosphorylated to give 4-phosphopantothenate by pantothenate kinase. This then reacts with cysteine to form 4-phosphopantothenylcysteine which is then decarboxylated to give 4-phosphopantetheine. The latter is then converted in two further steps to CoASH (Abiko, 1975). Here we have determined the substrate (pantothenate) and the product (4-phosphopantothenate) of pantothenate kinase in liver of mice bearing different tumours. We have also determined the activity of fatty acid synthetase in these livers since this enzyme contains a 4-phosphopantetheine residue.

### Materials and methods

Three to 4 month old male mice were used throughout. All animals were weight-matched, and fed a standard cubed diet with water *ad lib.* Where the effects of starvation were studied food, but not water was withdrawn for 24 h. TLX-5 lymphoma was maintained by regular i.p. passage in CBA strain mice every 5 days. Donor animals were killed by cervical dislocation and tumour cells harvested in Hank's Balance Salt Medium. Cell suspensions were diluted in the same medium and counted in a haemocytometer. Groups of 6 mice received $2 \times 10^6$ cells in 0.5 ml of medium either i.p. or s.c. in the subscapular region under light ether anaesthesia. Controls for these groups received 0.5 ml of the medium by the same routes. Sarcoma 180 was maintained in BALB/c mice. This tumour had been implanted 10 days previously s.c. in the subscapular region under light ether anaesthesia. Donor animals were killed by cervical dislocation, and the tumours dissected free from necrotic areas, then cut into small pieces (~2 mm) which were then inserted into groups of 6 mice by the same route, also under anaesthesia. A mouse fibrosarcoma in C57 mice was maintained in the same way, and by the same route in groups of recipients. Tumour-bearers and their corresponding controls were killed by cervical dislocation at the following times after implant: TLX-5 lymphoma, 6 days; sarcoma 180, 10 days and fibrosarcoma groups, 13 days. Livers were rapidly exposed, and clamped with tongs previously cooled in liquid nitrogen, then powdered in a mortar also cooled in liquid nitrogen. Two to three hundred mg powdered tissue was then placed in preweighed and precooled homogenising vessels. After a rapid reweighing, 5 ml of IM ice-cold perchloric acid was added, and the mixture homogenised then centrifuged (2,500 *g* for 10 min). The supernatants were removed, cooled in ice, and the pH adjusted to 6 with 2.5 M potassium carbonate. After further cooling in ice for 10 min followed by centrifugation (as above), aliquots of the supernatants were used for the determination of CoASH and acetyl CoA by a specific enzymatic method (Moellering & Bergmeyer, 1965). 'Total' CoA by this procedure, refers

**Figure 1** Biosynthesis of coenzyme A (CoASH).

Correspondence: R.A. McAllister.
Received 8 June 1987; and in revised form 4 August 1987.

to the sum of acetyl CoA and CoASH, plus the very small amounts of oxidised CoA present in liver.

For the assay of pantothenate, 100 to 200 mg frozen liver powder was homogenised with 0.9 ml of 8% perchloric acid–40% ethanol and 0.02 ml 1 M dithiothreitol. The mixture was centrifuged (2,500 g), the supernatants removed and cooled in ice. The pH was then adjusted to 7 with 1 M potassium carbonate, and after further cooling for 10 min, the perchlorate was removed by centrifugation (2000 g). Aliquots of the supernatants were heated in a boiling water bath for 1 min cooled and the pantothenate content determined (Skeggs & Wright, 1944) using lactobacillus plantarum (ATCC 8014). After 18 to 24 h growth in pantothenate assay medium (Difco Laboratories), turbidities were measured in a spectrophotometer at 600 nm. Standards containing 0 to 0.1 μg pantothenate were included with each batch, and were linear in this range. For the determination of 4-phosphopantothenate, aliquots of the neutralised PCA-ethanol extract were treated with prostatic acid phosphatase (Brown, 1959). The 'total' pantothenate was then assayed as above, and the amount of the 4-phospho derivative determined by difference. Similar groups of control and tumour-bearing animals were used for the separate determination of fatty acid synthetase activity in liver. Tissue was homogenised in 0.1 M phosphate buffer, pH 7, containing 0.1% by volume of mercaptoethanol, using a ratio of 1 g liver to 6 ml medium. The samples were then centrifuged at 40,000 g for 1 h. Aliquots of the supernatants, usually 0.04 ml were used for the assay by a spectrophotometric method (Lynen, 1962). The cyst(e)ine content of liver was determined using a specific ninhydrin method (Gationde, 1967) with prior incubation of neutralised perchloric acid extracts with dithiothreitol to convert cystine to cysteine. Statistical evaluation of results was determined as the mean plus or minus the standard error of the mean (SEM) and significance calculated on the basis of Student's $t$ test.

### Results

Growth of TLX-5 lymphoma, either i.p. of s.c. induced a significant increase ($P<0.001$) in the levels of both pantothenate and 4-phosphopantothenate (Figure 2). In contrast, in mice bearing either sarcoma 180, or the fibrosarcoma, the pantothenate levels in liver decreased significantly, but in both models the 4-phosphopantothenate content showed significant increases (Figure 3). As can be seen (Table I) in starved normal mice the pantothenate content fell to zero, without any significant change in the 4-phosphopantothenate content. It should be noted, however, that the sensitivity of the method for pantothenate is ~0.01 μg.

In agreement with our previous studies (McAllister *et al.*, 1982), growth of TLX-5 lymphoma i.p. induced significant decreases in 'total' CoA, acetyl CoA and CoASH. In the present study, mice growing this tumour s.c. also showed significant decreases in the levels of these metabolites (Table II). Also shown are the decreased levels in mice bearing either Sarcoma 180, or the fibrosarcoma.

The cysteine content of liver of mice bearing the different tumours did not alter significantly from the corresponding controls. Values found here for normal mice were 0.094 μmol $g^{-1}$ liver wet weight ± s.e.m. 0.037, with little or no cystine present.

In mice growing TLX-5 i.p. there were highly significant decreases in the activity of fatty acid synthetase in liver. A significant decrease ($P<0.01$) was also found in mice bearing the fibrosarcoma (Figure 4). In contrast growth of sarcoma 180 induced a significant increase ($P<0.001$). It will be noted from the data (Figure 4) that the activity of the enzyme in liver of normal CBA mice (control group for mice bearing TLX-5 lymphoma) is considerably higher than in normal BALB/c or C57/B/6J mice, which were controls for the sarcoma and fibrosarcoma groups respectively. We have checked this on several occasions and can only attribute the difference to some unknown factor in the strain of mice used.

**Figure 2** The pantothenate and 4-phosphopantothenate of normal control mice (c) and mice bearing TLX-5 lymphoma (T) either i.p. or s.c. Values are mean ± s.e. of 6 animals in each group. ***$P<0.001$.

**Figure 3** The pantothenate and 4-phosphopantothenate content of livers of normal control mice and mice bearing either sarcoma 180 or a fibrosarcoma (FMT 138). Values are means ± s.e. of 6 animals in each group. ** = $P<0.01$; *** = $P<0.001$.

### Discussion

Coenzyme A plays a central role in fatty acid and pyruvate oxidation, and is the precursor of the 4-phosphopantetheine residue of fatty acid synthetase (Abiko, 1975). The pathway

**Table I** The effect of fasting for 24 h on the levels of pantothenate and 4-phosphopantothenate in mouse liver[a]

| *Metabolic state* | *Pantothenate* | *4-Phosphopantothenate* |
|---|---|---|
| Normal fed. (6) | 2.48±0.12 | 4.13±0.23 |
| Starved 24 h (6) | n.d. | 4.95±0.38 |

[a]The numbers in parentheses give the number of CBA mice in each group. Values are means±s.e. of duplicate assays and are in terms of $nmol\,g^{-1}$ liver wet wt; n.d. = none detected; limit of determination of method used is ~0.01 μg pantothenate.

**Table II** The effect of tumour growth in mice on the hepatic contents of acetyl CoA, CoASH and 'total' CoA

| *Tumour* | *Acetyl CoA* | *CoASH* | *'Total' CoA* |
|---|---|---|---|
| Control | 75.63±7.76 | 77.31±6.27 | 152.87±5.12 |
| TLX-5 i.p. | 37.34±4.10 | 25.11±1.50 | 62.46±5.54 |
| | $P$<0.001 | $P$<0.001 | $P$<0.001 |
| Control | 77.81±3.28 | 71.89±2.79 | 149.70±5.03 |
| TLX-5 s.c. | 51.85±2.47 | 47.74±4.16 | 99.59+3.24 |
| | $P$<0.01 | $P$<0.001 | $P$<0.001 |
| Control | 77.10±2.37 | 77.44±5.49 | 154.54±6.10 |
| Sarcoma 180 s.c. | 53.98±3.59 | 62.14±2.81 | 126.14±2.97 |
| | $P$<0.02 | $P$<0.05 | $P$<0.01 |
| Control | 70.25±3.10 | 74.36±4.89 | 144.61±4.22 |
| FMT s.c. | 54.19±2.20 | 53.92±4.08 | 108.11±3.20 |
| | $P$<0.01 | $P$<0.01 | $P$<0.001 |

Six animals were studied in each group. Values are for mean±s.e. and are expressed in $nmol\,g^{-1}$ liver wet wt.

**Figure 4** Fatty acid synthetase activity in liver of normal control mice and mice brearing TLX-5 lymphoma i.p. or s.c.; sarcoma 180 or a fibrosarcoma (FMT 138). C = controls; T = tumour-bearers. Values are means±s.e. of 6 animals in each group. ** = $P$<0.01; *** = $P$<0.001. One unit is the amount of enzyme which under the condition of the assay oxidises 1 μmol NADPH $min^{-1}$ (corresponding to 0.5 μmol malonyl CoA or a change in extinction of 0.004).

of CoA synthesis is known, and it has been shown that pantothenate kinase may be the regulatory enzyme in this pathway, since it is subject to feedback inhibition by CoA (Abiko, 1975), and its activity can be increased by drugs causing increases in the CoA content of tissues (Skrede & Halvorsen, 1979). The small, but significant increases in 4-phosphopantothenate concentrations accompanied by small but significant increases in pantothenate in livers of mice bearing either sarcoma 180, or the fibrosarcoma indicate an increased activity of pantothenate kinase. The reduction in the hepatic content of CoA in mice bearing sarcoma 180, could give rise to this increased activity as there will be a reduction in the CoA inhibition of pantothenate kinase. Large increases in both pantothenate and 4-phosphopantothenate were found in mice bearing TLX-5 lymphoma either s.c. or i.p. Since we have shown previously (McAllister *et al.*, 1982), that there are marked reductions in the food intake of mice bearing this tumour i.p., and Smith *et al.* (1978) have reported increases in pantothenate in liver of starved rats, we examined the effect of starvation in normal mice on the hepatic content of pantothenate, and 4-phosphopantothenate. The level of pantothenate fell to zero in these livers with no change in 4-phosphopantothenate. The large increases in both of these metabolites in mice bearing the lymphoma either s.c., or i.p. are in contrast to the results found with sarcoma 180 or the fibrosarcoma. These large increases could be due to either a large reduction in the rate of conversion of a later intermediate in the pathway to CoA, or a very much larger production of pantothenate and 4-phosphopantothenate from the degradation of CoA, or possibly the 4-phosphopantetheine residue of fatty acid synthetase. The reduction in the hepatic content of 'total' CoA in mice bearing TLX-5 lymphoma is of the order of 80 $nmol\,g^{-1}$ liver wet weight when the tumour is i.p. and this reduction is more than sufficient to account for the ~40 nmol increase in total pantothenate and 4-phosphopantothenate found in these animals. Changes in the levels of acetyl CoA and CoASH reported here, are in agreement with our previous findings (McAllister *et al.*, 1982; 1984). Since the cysteine content of liver did not alter in the presence of the tumour showed that these changes in CoA did not depend upon the availability of cysteine. The degradation of CoA in liver is nearly a reversal of the biosynthetic route. The initial step is the conversion of CoA to dephosphocoenzyme A by lysosomal, acid phosphatase, and the final step the production of pantothenate from pantetheine catalysed by pantethinase, which is found in the microsomal–lysosomal fraction (Abiko, 1975). Since increased activity of lysosomal enzymes have been reported in tumours, and liver of tumour-bearing animals (Ferguson *et al.*, 1979) it is possible that this is responsible for the increased dissimilation of CoA in mice bearing TLX-5 lymphoma.

CoA is the precursor of 4-phosphopantetheine and recent estimates of the 4-phosphopantetheine content of the enzyme give values of ~2 moles of 4-phosphopantetheine per mole of enzyme (Qureshi *et al.*, 1975). Thus the activities of fatty acid synthetase found represent, 7.5–10.1 nmol 4-phosphopantetheine $g^{-1}$ wet weight of liver in CBA controls, and 2.4 and 2.6 $nmol\,g^{-1}$ wet weight in BALB/c and C57/B/6J mice respectively. The small variations in fatty acid synthetase activity found in liver of the latter strain of mouse bearing the fibrosarcoma represent changes of phosphopantetheine content of about 0.5 $nmol\,g^{-1}$. The large changes in activity found in mice bearing TLX-5 lymphoma represent changes in phosphopantetheine content of 6.5 and 7.3 $nmol\,g^{-1}$. Thus the 4-phosphopantetheine prosthetic group of fatty acid synthetase can only contribute a small amount to any increase in cellular levels of pantothenate and 4-phosphopantothenate. However, the reduction in fatty acid synthetase activity in mice bearing TLX-5; may be a reflection of a reduced cytosolic CoA concentration which is needed for the conversion of apoenzyme to the holo-enzyme (Qureshi *et al.*, 1975). This is unlikely to be the sole reason, as mice bearing sarcoma 180 or the fibrosarcoma both show reduced CoA contents, but only small changes in fatty acid synthetase activity, and pantothenate and 4-phosphopantothenate concentrations. It is known that the synthesis of fatty acid synthetase is regulated by hormones such as insulin and triiodothyronine (Lornitzo *et al.*, 1981), and if only TLX-5 lymphoma affected levels of these hormones then this could explain the differential effects of this tumour.

At present we have no information on the level of circulating insulin in these animals, but in rats bearing the Walker 256 carcinoma, it has been reported that insulin levels are significantly decreased (Goodlad *et al.*, 1975).

We are aware that in studies such as the present one, the use of rapidly growing murine tumours may bear little resemblance to actual changes which occur in humans with slow growing tumours. However, previously we showed that in mice bearing a slow growing mammary tumour there were marked decreases in the levels of CoASH and acetyl CoA in liver. These changes occurred in animals in which the mean tumour weight was 16.3 mg $\pm$ s.d. 0.003, and only just palpable *in situ*. The tumour having been implanted 14 days previously and represented 0.06% of the host mass (McAllister *et al.*, 1982).

A further problem in such studies is the provision of suitable controls. In earlier work (McAllister, 1978) normal CBA mice were injected with spleen cells from the same strain of mice to serve as controls for those bearing the lymphoma. No changes however occurred from normal in the levels of CoASH and acetyl CoA in liver following this treatment.

Both animal and human malignancies are known to affect the vitamin status of the host (Dickerson, 1983). These changes include uptake of the vitamin by the tumour (Anthony & Schorah, 1982), and alterations in the metabolism of the vitamin by the tumour (Rivlin, 1973). CoA is the metabolically-active form of pantothenate and our data suggest that growth of these tumours affects pantothenate metabolism in the host. Of interest is the observation of Aptekar and Ganetskaia (1965) on riboflavin. They showed that in mice bearing sarcoma the riboflavin and riboflavin phosphate levels in liver remained normal, but the level of flavin adenine dinucleotide, the metabolically active form of riboflavin fell significantly.

We thank Dr H.G. Nimmo for a gift of rat liver fatty acid synthetase.

### References

ABIKO, Y. (1975). Metabolism of coenzyme A. In *Metabolic Pathways*, **8**, Greenberg, D.M. (ed) p. 1. Academic Press: New York.

ANTHONY, H. & SCHORAH, C. (1982). Severe hypovitaminosis C in surgical repair and lymphocyte-related host resistance. *Br. J. Cancer*, **46**, 354.

APTEKAR, S.G. & GANETSKAIA, S.A. (1965). Riboflavin content in organs of mice with transplantable tumours. *Arkh. Patol.*, **27**, 63.

BALDUCCI, L. & HARDY, C. (1985). Cancer and malnutrition – A critical interaction. A review. *Amer. J. Haematol.*, **18**, 91.

BROWN, G.M. (1959). Assay and distribution of bound forms of pantethenic acid. *J. Biol. Chem.*, **234**, 379.

DICKERSON, J.W.T. (1983). Nutrition of the cancer patient. *Adv. Nutr. Res.*, **5**, 106.

FERGUSON, W.W., FIDLER, M.R., FOLKMAN, C.K. & STARLING, J.R. (1979). Correlation of lysosomal enzymes and cachexia in the tumour-bearing rat. *J. Surg. Res.*, **26**, 150.

GATIONDE, M.K. (1967). A spectrophotometric method for the direct determination of cysteine in the presence of other naturally occurring amino acids. *Biochem. J.*, **104**, 627.

GOODLAD, G.A.J., MITCHELL, A.J.H., McPHAIL, L. & CLARK, C.M. (1975). Serum insulin and somatomedin levels in the tumour-bearing rat. *Eur. J. Cancer*, **11**, 733.

KRALOVIC, R.C., ZEPP, A. & CENEDELLA, R.J. (1977). Studies on the mechanism of carcass fat depletion in experimental cancer. *Eur. J. Cancer*, **13**, 1071.

LORNITZO, F.A., KATIYAR, S.S., PURI, R.N. & PORTER, J.W. (1981). Demonstration of the occurrence of inactive fatty acid synthetase in rat liver by immunotitration and it *in vitro* partial activation. *J. Biol. Chem.*, **256**, 8498.

LYNEN, F. (1962). Fatty acid synthetase from malonyl CoA. In *Methods in enzymology*, **5**, Colowick, S.P. & Kaplan, N. (eds) p. 443. Academic Press: New York.

McALLISTER, R.R. (1978). *Metabolic Changes in Tumour Bearing Animals*. Ph.D thesis, University of Glasgow, p. 33.

McALLISTER, R.A., CALMAN, K.C. & CAMPBELL, E.H.G. (1982). Metabolic changes in liver of tumour-bearing mice. *J. Surg. Res.*, **33**, 500.

McALLISTER, R.A. & CAMPBELL, E.H.G. (1982). Changes in the hepatic content of long-chain acyl CoA in liver of tumour-bearing mice. *IRCS Med. Sci.*, **10**, 947.

McALLISTER, R.A. & CAMPBELL, E.H.G. (1984) The effect of tumour site on the rate of lipolysis in epididymal adipose tissue of the mouse. *IRCS Med. Sci.*, **12**, 1018.

MOELLERING, H. & BERGMEYER, H.U. (1965). Kinetic determination of CoA. In *Methods of enzymatic analysis*, **4**, Bergmeyer, H.U., (ed) p. 1811. Academic Press: New York.

QURESHI, A.A., KIM, M., LORNITZO, F., JENIK, R.A. & PORTER, J.W. (1975). Separation of pigeon liver apo- and holo-fatty acid synthetase by affinity chromatography. *Biochem. Biophys. Res. Comm.*, **64**, 835.

RAPP, G.W. (1973). Some systemic effects of malignant tumours 1. Coenzyme A levels. *Cancer*, **31**, 357.

RIVLIN, R.A. (1973). Riboflavin and cancer. A review. *Cancer Res.*, **33**, 1977.

SHAPOT, V.S. & BLINOV, V.A. (1974). Blood glucose levels in animals bearing transplantable tumors. *Cancer Res.*, **34**, 1827.

SKEGGS, H.R. & WRIGHT, L.D. (1944). The use of lactobacillus arabinosus in the microbiological determination of pantothenic acid. *J. Biol. Chem.*, **156**, 21.

SKREDE, S. & HALVORSEN, O. (1979). Increased biosynthesis of CoA in liver of rats treated with clofibrate. *Eur. J. Biochem.*, **98**, 223.

SMITH, C.M., CANO, M.L. & POTYRAJ, J. (1978). The relationship between metabolic state and total CoA content of rat liver and heart. *J. Nutr.*, **108**, 854.